# Polygenic risk modeling of tumor stage and survival in bladder cancer

**DOI:** 10.1186/s13040-022-00306-w

**Published:** 2022-09-30

**Authors:** Mauro Nascimben, Lia Rimondini, Davide Corà, Manolo Venturin

**Affiliations:** 1 Department of Health Sciences, Università del Piemonte Orientale, Via Solaroli 17, 28100 Novara, Italy; 2 Enginsoft SpA, Via Giambellino 7, 35129 Padova, Italy; 3 Department of Translational Medicine, Università del Piemonte Orientale, Via Solaroli 17, 28100 Novara, Italy

**Keywords:** Data-driven biomarker research, Polygenic risk modeling, Non-linear dimension reduction, Tree ensemble embedding

## Abstract

**Introduction:**

Bladder cancer assessment with non-invasive gene expression signatures facilitates the detection of patients at risk and surveillance of their status, bypassing the discomforts given by cystoscopy. To achieve accurate cancer estimation, analysis pipelines for gene expression data (GED) may integrate a sequence of several machine learning and bio-statistical techniques to model complex characteristics of pathological patterns.

**Methods:**

Numerical experiments tested the combination of GED preprocessing by discretization with tree ensemble embeddings and nonlinear dimensionality reductions to categorize oncological patients comprehensively. Modeling aimed to identify tumor stage and distinguish survival outcomes in two situations: complete and partial data embedding. This latter experimental condition simulates the addition of new patients to an existing model for rapid monitoring of disease progression. Machine learning procedures were employed to identify the most relevant genes involved in patient prognosis and test the performance of preprocessed GED compared to untransformed data in predicting patient conditions.

**Results:**

Data embedding paired with dimensionality reduction produced prognostic maps with well-defined clusters of patients, suitable for medical decision support. A second experiment simulated the addition of new patients to an existing model (partial data embedding): Uniform Manifold Approximation and Projection (UMAP) methodology with uniform data discretization led to better outcomes than other analyzed pipelines. Further exploration of parameter space for UMAP and t-distributed stochastic neighbor embedding (t-SNE) underlined the importance of tuning a higher number of parameters for UMAP rather than t-SNE. Moreover, two different machine learning experiments identified a group of genes valuable for partitioning patients (gene relevance analysis) and showed the higher precision obtained by preprocessed data in predicting tumor outcomes for cancer stage and survival rate (six classes prediction).

**Conclusions:**

The present investigation proposed new analysis pipelines for disease outcome modeling from bladder cancer-related biomarkers. Complete and partial data embedding experiments suggested that pipelines employing UMAP had a more accurate predictive ability, supporting the recent literature trends on this methodology. However, it was also found that several UMAP parameters influence experimental results, therefore deriving a recommendation for researchers to pay attention to this aspect of the UMAP technique. Machine learning procedures further demonstrated the effectiveness of the proposed preprocessing in predicting patients’ conditions and determined a sub-group of biomarkers significant for forecasting bladder cancer prognosis.

## Introduction

Machine learning (i.e., ML) and bio-statistics offer a wide range of methodologies to build models able to estimate several aspects of cancer from gene expression data (i.e., GED). A distinguishing feature of machine learning models is that they afford to predict from data rather than infer, a typical paradigm of statistics [1]. Predictive models that contextualize disease risk by accounting for the heterogeneity of changes in patient bodies lead to individual-specific medicine. This emerging branch of medical science is called precision or personalized medicine aiming to produce preventative strategies to tackle illnesses [2]. Genomics, and more in general, omics techniques, offer large amounts of data to assess the risk of disease progression. In cancer, genomics can reveal molecular underpinnings and provide insights into possible targets for future therapies. However, biorepositories should provide standardized and quality samples for ML models to capture the significant individual genetic variants found between human populations and accomplish proper individual-based diagnosis and prognosis [3, 4]. Polygenic risk models gather contributions from a set of genes to create a single model capable of summing up the complexity of the different biological changes connected with a disease [5]. When single markers cannot provide proper support to construct risk prediction scores, gene ensembles can summarise genetic effects more accurately. However, polygenic datasets aggregate data in high-dimensional spaces sparser than those built in lower dimensions, thus suffering from geometric distortion [6]. Consequently, the high-dimensionality of GED data could negatively impact the generalization ability of standard machine learning methods, impairing the scalability and interpretability of the model. The association of different ML and bio-statistical sequential methods in bioinformatics data analysis workflows offers the possibility of modeling biological processes, overcoming linear and parametric approaches limitations, and transforming raw gene expression values into helpful information for clinicians.

### Aim of the study

Previous work introduced double discretization procedures to characterize GED applicable when modeling bladder cancer survival rate (supervised binary classification) [7]. The present manuscript employed the same dataset of bladder cancer biomarkers, integrating initial numerical discretizations into a new bioinformatics framework. It enclosed forest embedding and manifold dimensionality reduction to produce graph-like forecasts exposing peculiar patterns suitable for extending patient categorization into six classes (three grades of tumor severity and two classes for overall survival) in an unsupervised fashion. The inclusion of cancer staging supports medical decisions regarding prognosis and treatment. Multiple numerical experiments will analyze and evaluate different aspects of the proposed procedure and the obtained results.

## Methods

**Fig. 1:**
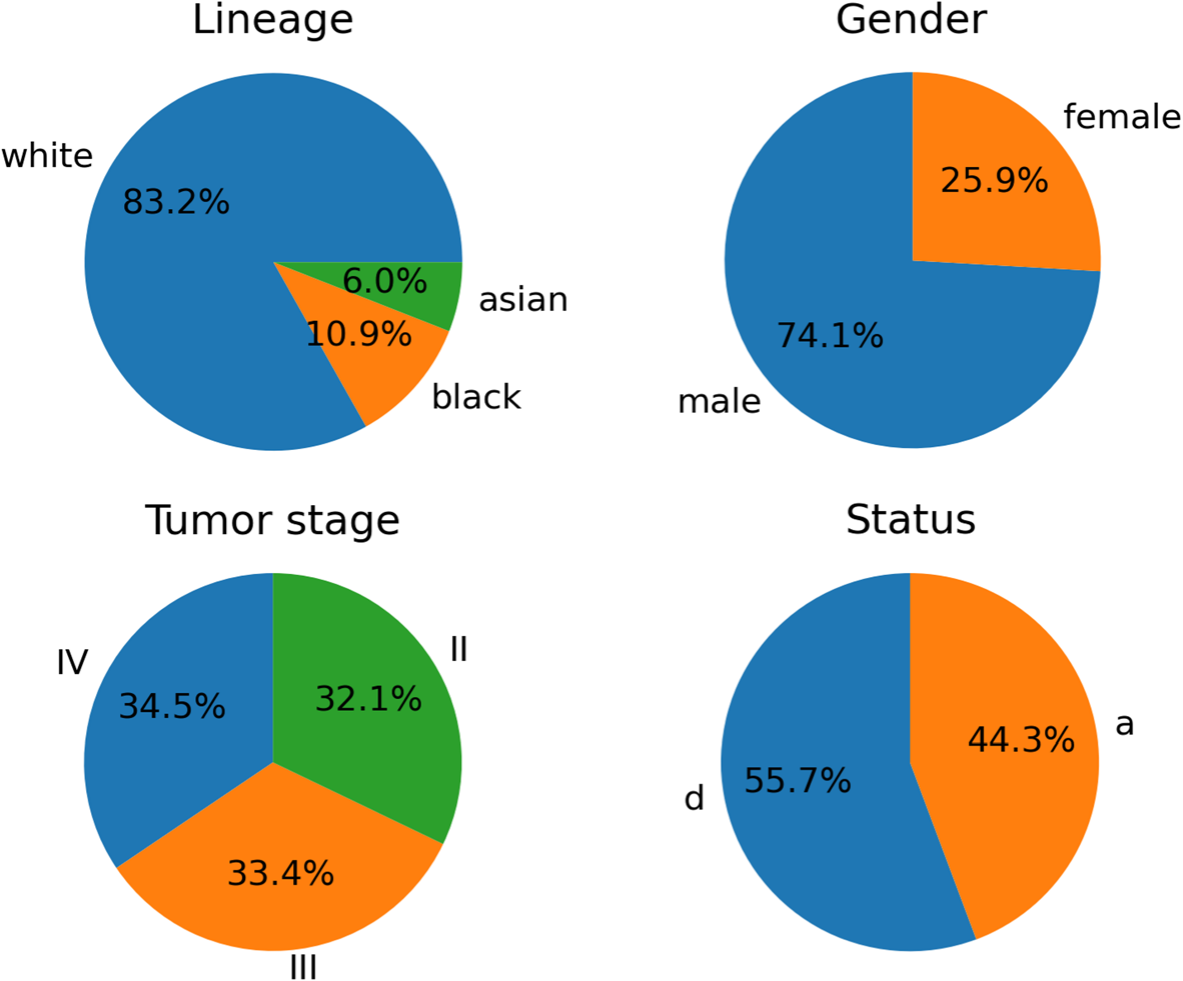
Descriptive information of the cohort of patients included in the dataset. In clockwise order, the pie charts show in the top left corner the lineage, the percentage of males or females, the rate of patients dead or alive, and the tumor stage at the time of data collection

**Fig. 2:**
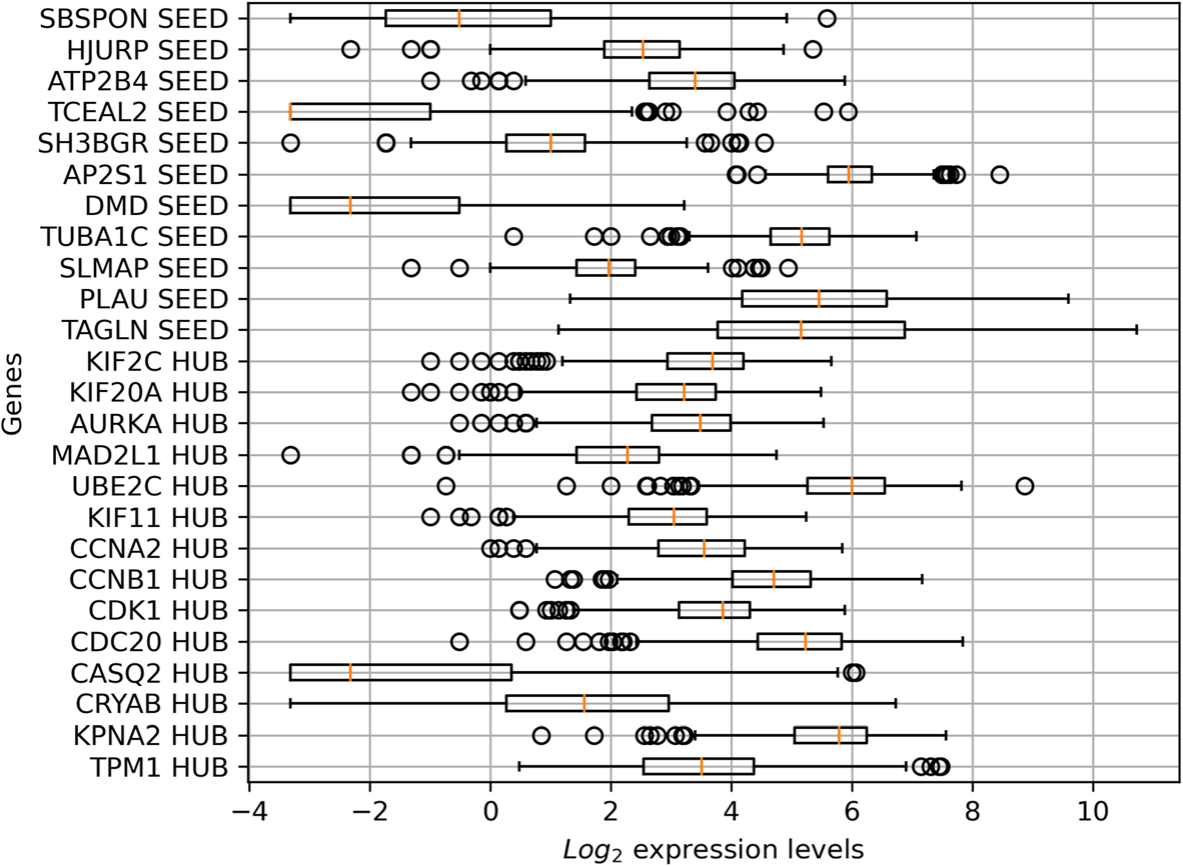
The boxplots depict $log_{2}$ expression levels for the hub and seed genes before preprocessing

**Fig. 3:**
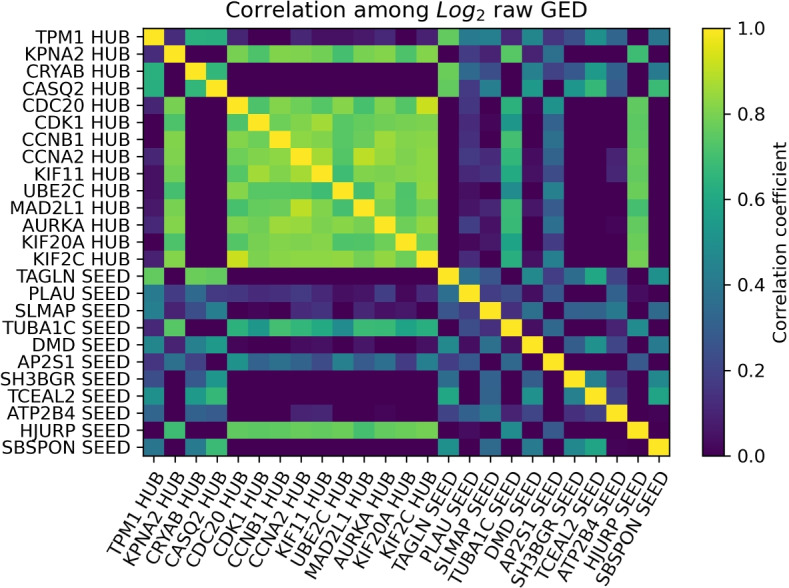
The heatmap reports the Pearson product-moment correlation coefficients of $\log _{2}$ expression levels for the hub and seed genes before preprocessing

**Fig. 4:**
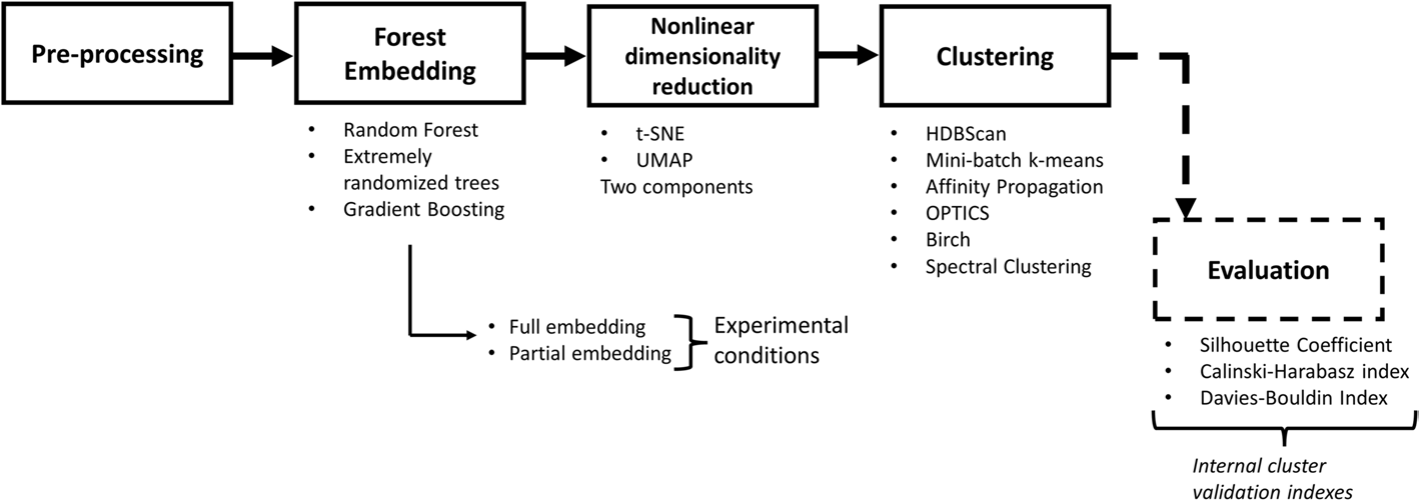
Outline of the analysis pipeline to produce complete and partial forest embeddings

Gene expression data were collected by [8] and released as a public domain data file. Authors evaluated genes related to bladder cancer and selected those most active during different stages of the disease. They identified 14 hub genes, genetic buffers highly connected with others showing augmented genetic interaction, and also defined 11 seed genes, likewise the procedure of [9]. Seed genes were identified from the relevant subnetworks recognized with FUNRICH software [10] by molecular complex detection analysis. Genetic profiles of the hub and seed genes came from 406 patients, but 20 subjects could not be labeled, and they were removed because they had multiple missing entries for tumor stage or survival outcome. The patient’s descriptive information was added in Fig. 1, while the raw $log_{2}$ expression levels were included in Fig. 2, with the correlation among genes in Fig. 3. Log-transformation of raw gene expression data is usually accomplished to compensate for data skewness and approximate a normal distribution. Indeed, data showed a prominent right skewness treated applying deterministic mathematical functions during preprocessing. Generally, this step is accomplished to fulfill the assumptions of parametric inference, but it also helps learn and generalize specific ML models [11, 12]. All analysis was carried out with custom scripts in Python programming language, partly employing umap-learn [13], imbalanced-learn [14], and scikit-learn libraries [15]. An overview of the whole experimental sequence is shown in Fig. 4.

### Preprocessing

Three alternative discretization approaches derived from the procedure previously investigated in [7] constituted the initial phase of the data handling scheme:

“Log-z”: Each GED was log-transformed and values standardized. Continuous values were discretized by Classification and Regression Trees (i.e., CART [16]).
“Uniform”: Cumulative distribution function of each GED was estimated and, through a quantile function, mapped to a uniform distribution with data normalization in the interval [0;1]. The number of quantiles (51) introduced a pre-binning of the data followed by CART discretization after uniform mapping.
“Normal”: Cumulative distribution function of each GED was estimated through a quantile function mapped to a normal distribution with data standardization. The number of quantiles (51) introduced a pre-binning of the data followed by CART discretization after normal mapping.

**Fig. 5:**
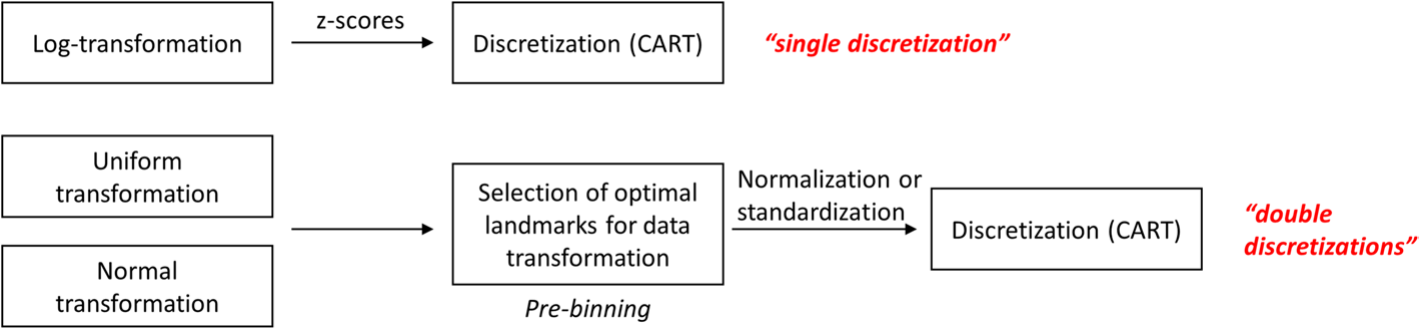
Overview of initial GED discretizations applied as preprocessing

It should be noted that Uniform and Normal data mapping produced a “double discretization” on data as demonstrated in the previous exploration: operational sequences are synthesized in Fig. 5. In general, discretization transforms values in intervals acting as a variable selection that benefits classification [17]. Usage of CART is not an arbitrary decision but offered remarkable performance during the earlier analysis run on the same data. Afterward, discretized GED was labeled in six categories, generated from tumor stage (II, III, IV) and disease outcome (alive or dead, abbreviated as “a” or “d” respectively). This multiclass problem poses more challenges than the previously considered models targeting survival binary classification. A critical issue in multi-label classification is the skewness of the labels, also called class imbalance, the biased distribution of examples across the known classes [18]. When classes are not equally represented, introducing new “synthetic” values could be a way to aid learning [19]. Re-balancing training set values by over-sampling or under-sampling is equivalent to altering the misclassification cost ratio and has little effect on Bayesian or decision tree learning methods [20]. Nonetheless, using sampling for cost-sensitivity learning has a few disadvantages, like dropping potentially profitable data or enlarging dataset size [21]. A methodology proposing a compromise to reduce sampling drawbacks is the Synthetic Minority Over-Sampling Technique [22] (i.e., SMOTE). Here we enhanced SMOTE by pairing it with the Tomek link algorithm [23]. While SMOTE induced new synthetic minority class examples, Tomek links ensure the removal of sample pairs nearest neighbors belonging to two different classes. It happens when interpolated minority class examples invade the majority class space. Pre-processing data with these two algorithms in sequence mitigates the effect of over-sampling by removing noisy values too close to the optimal decision boundary [24], leading to more defined class clusters during training (Table 1).

**Table 1:** Number of examples in each class after preprocessing

	IIa	IIIa	IVa	IId	IIId	IVd	Total
Original dataset	88	80	47	36	53	82	386
Log-z	69	72	87	87	82	77	474
Uniform	73	75	82	84	80	70	464
Normal	75	73	84	86	82	76	476

### Tree ensemble embedding

Trees are hierarchical structures, starting with a root value (a feature or GED) and descendant sub-trees generated from parent nodes: each split into branches is called edge. The end of a branch is called a leaf. The characteristics of trees are the existence of exactly one path (connected sequence of edges) between any pair of nodes and their acyclicity because there is no loop in their topology. Large numbers of trees operating as an ensemble are called forests. Feature spaces could be represented by forest embedding, collecting leaf value sequence for each observation to obtain a similarity matrix resembling the procedure applied by [25] on prostate tumor marker data with random forests. Other successful tumor marker profiling with forest embedding could be found in [26], where authors explored random forest proximity matrix as input measure for clustering algorithms, or in [27] for genomic data analysis. Within a multitude of decision trees, the similarity is computed by counting the number of times observations from different trees fall in the same leaf, normalizing the results by the total number of trees: the assumption is that feature points closer to each other will enter in the same leaf. In the current work, three possible tree ensembles were evaluated to build the proximity matrix: random forest [28], gradient boosting [29], and extremely randomized trees [30], all verified by 10-fold stratified cross-validation. Extremely randomized trees produce trees less correlated than random forests, while gradient boosting also combines decision trees but builds one tree at a time linking results during the process (not at the end by averaging as random forests do). For all three preprocessing transformations, the optimal number of trees was selected by grid search, balancing class instances by weighting their frequency as a penalization parameter and employing balanced accuracy as a comparison metric. The foremost model was the extremely randomized trees, therefore selected to build the proximity matrices in the two experimental conditions.

### Dimensionality reduction

Dimensionality reduction produces a representation that helps identify relevant data patterns. For example, in [25], the authors applied multi-dimensional scaling to expose the peculiar structure of point clouds for each class in bi-dimensional space. Two different methodologies were compared in the present work: heavy-tailed t-distributed stochastic neighbor embedding (i.e., t-SNE) [31] or uniform manifold approximation and projection (i.e., UMAP) [32]. Stochastic neighbor embedding computes the probability distribution over pairs of points in both original data (high dimensional dataset) and low dimensional embedding space, minimizing the Kullback-Leibler divergence between probability distributions (usually performed using gradient-descent techniques), producing the low dimensional embedding. During the numerical experiments of this research, the Barnes-Hut SNE implementation was chosen due to its computational efficiency [33]. UMAP algorithm constructs a topological representation (fuzzy simplicial sets) of data approximated through the medium of Riemannian manifolds both for high and low dimensional spaces. Then the low dimensional space representation is optimized by minimizing fuzzy set cross-entropy via stochastic gradient descent to reduce the error between representations. Both t-SNE and UMAP produce maps of point clouds convenient to categorize GED in sub-populations and highlight significant differences between groups. However, hyperparameter tuning is not trivial for both algorithms. For this reason, during the dimensionality reduction phase, an extensive examination of the best parameters was attempted as a combination of grid and random search in hyperparameter space (studied parameters arranged in Tables 2 and 3). Another essential aspect is given by the nature of t-SNE that does not preserve the global geometry of the data even if it produces isolated groups attractive as input for clustering algorithms. To mitigate the arbitrary position effect of cloud points created by the algorithm in the embedded space, t-SNE was initialized with principal component analysis and learning rate included as a hyperparameter to be tuned (generally increased). In addition number of iterations was set to 3000 to enhance visualization, as suggested in [34]. In both t-SNE and UMAP, different metrics for calculating distance between instances were attempted because euclidean distance alone may not be adequate in multi-dimensional feature spaces [35]. For instance, the nearest neighbor concept is ill-defined as points become uniformly distant from each other [36]. A further issue with t-SNE is adding new data to the embedding already learned; in its original form, t-SNE is a non-linear, non-parametric embedding that requires re-learning the whole dataset when appending unseen points. Rather than t-SNE, UMAP preserves global data structure and allows new data transformation into the learned space. In our partial embedding experiment, we are not adding new data to t-SNE or UMAP, as they continue to learn the whole dataset, but the tree ensemble embedding is achieved on a sub-sample of the dataset, then mapped in lower-dimensional space.

**Table 2:** t-SNE parameters

Parameter	Abbreviation	Levels
Angular size for Barnes-Hut	$\theta$	8
Early exaggeration	EE	8
Learning rate	LR	14
Metric for distance between instances	Metr	9
Perplexity	Perp	11

**Table 3:** UMAP parameters

Parameter	Abbreviation	Levels
Learning rate	LR	8
Metric for high dimensional space distances calculation	Metr	8
Number of nearest neighbors assumed at local level	LC	5
Dispersion of points on manifold	MiD	5
Size of neighboring sample points in manifold estimation	NN	6
During optimization, ratio of negative samples per positive example	NSR	3
Negative samples penalization while optimizing in low dimension	RS	4
Ratio of fuzzy set operations to obtain global fuzzy simplicial sets	Mix	5
Spread out scale of embedded points	Sp	5

### Clustering

Several clustering techniques were implemented to sub-divide the dimensionally reduced forest embedding matrix and assess the goodness of the resulting bi-dimensional maps: hierarchical density-based spatial clustering of applications with noise (i.e., hdbscan [37]), mini-batch k-means [38], spectral clustering (i.e., SC [39]), ordering points to identify the clustering structure (i.e., optics [40]), affinity propagation (i.e., AP [41]), balanced iterative reducing and clustering using hierarchies (i.e., birch [42]). Multiple algorithms were taken into account because, as stated by the “no free lunch theorems”, algorithm selection is problem-specific, and there are no generally superior algorithms [43]. Among those considered, few algorithms (for example, mini-batch k-means) required defining a predetermined number of clusters as an input parameter. In such a case, the elbow method was implemented to decide the number of clusters in the data. The performance of clustering algorithms was determined by *internal cluster validation indexes* like Davies-Bouldin index (i.e., DBI) [44], silhouette score [45], and Calinski-Harabasz index (i.e., CHI) [46]. Internal metrics catch separation (spacing between different groups) and, at the same time, compactness (points density inside each group) of clusters. A custom method that maximizes silhouette score and CHI while minimizing DBI was calculated to identify the best algorithm and parameter combination for dimensionality reduction and clustering. High CHI values mean dense and well-separated clusters, while a high silhouette coefficient implies appropriate grouping, with values adequately assigned. A small DBI embodies the concept that clusters are distant and compact. The customized methodology was performed by selecting the occurrence with the smallest mean difference from $max{\left( Silhouette\;score\right) }$, $min{\left( DBI\right) }$, and $max{\left( CHI\right) }$. During this operation, values of CHI were scaled in the range 0-1 to match the range of values of DBI and silhouette score. As a final check, the absence of the noise label introduced by certain clustering algorithms was verified; otherwise, the result was discarded, taking the next value in rank. The *External cluster validation methods* have been estimated for the final comparison between pipelines. Validation indicators included Fowlkes-Mallows index [47], Rand index adjusted for chance [48], adjusted mutual information between two clusterings to account for chance [49], normalized mutual information [50], homogeneity and completeness metrics of a cluster labeling together with their harmonic mean (also called v-measure) [51]. External validation metrics appraise clustering labels compared to ground truth. Fowlkes-Mallows index metric judges similarity of the clusters with values ranging from zero (random groups) to one (exact classification). Mutual information-derived metrics evaluate the entropy reduction obtained if a class label is assigned to the right group based on absolute and conditional probabilities related to class membership. The adjusted Rand and mutual information update the indexes account for agreement solely due to chance; the former is more suitable for clusters of similar size while the latter can gauge unbalanced groups, a situation where the Rand index might be biased. Homogeneity of a partition considers if groups are uniform in their composition, while completeness checks if all class instances are assigned correctly. Both are desirable features during clustering and do not require assumptions regarding the cluster’s structures but might suffer random assignment to groups as they are not adjusted for the chance. Eventual label permutations do not influence the v-measure index being the harmonic mean between homogeneity and completeness. It is considered a more comprehensive measure of homogeneity and completeness and considers all data instances independently from cluster sizes or the number of clusters.

### Experimental conditions

The investigation was subdivided into two experimental conditions, each with a different tree ensemble embedding. The first condition was a complete embedding of the GED by the tree ensemble from which t-SNE and UMAP generated a bi-dimensional prognostic map, revealing cancer patients’ population patterns. The second condition was a partial embedding obtained by training the tree ensemble on 75% of the data. This situation simulates the addition of 25% unseen patients to an existing model to verify the behavior of each analysis pipeline under extreme circumstances. Indeed, it is unlikely that the model will categorize a large cohort of patients all at once; consequently, the second experimental condition could be interpreted as a “stress test” to check model reliability compared to the baseline condition of fully embedded data.

## Results

The results of the numerical experiments on the dataset could be summarized into five main findings:

- Demonstration of how the proposed analysis sequence leads to the creation of bi-dimensional prognostic maps to support medical decision-making (complete embedded experimental condition)
- Evaluation of a partial GED embedding to simulate the addition of new patients to an existing forest embedding (partial embedding experimental condition)
- Investigation of the parameter space for t-SNE and UMAP to highlight those that impact the low dimensional embedding and should be tuned when employing these techniques
- Test the performance of a classification model on six classes of tumor outcomes using the original GED set ($log_{2}$ transformed) or preprocessed GED by single and double discretization approaches
- Machine learning–based gene relevance analysis to ascertain the existence of a subset of genes remarkably involved in determining disease’s states

### Complete and partial forest embeddings

**Fig. 6:**
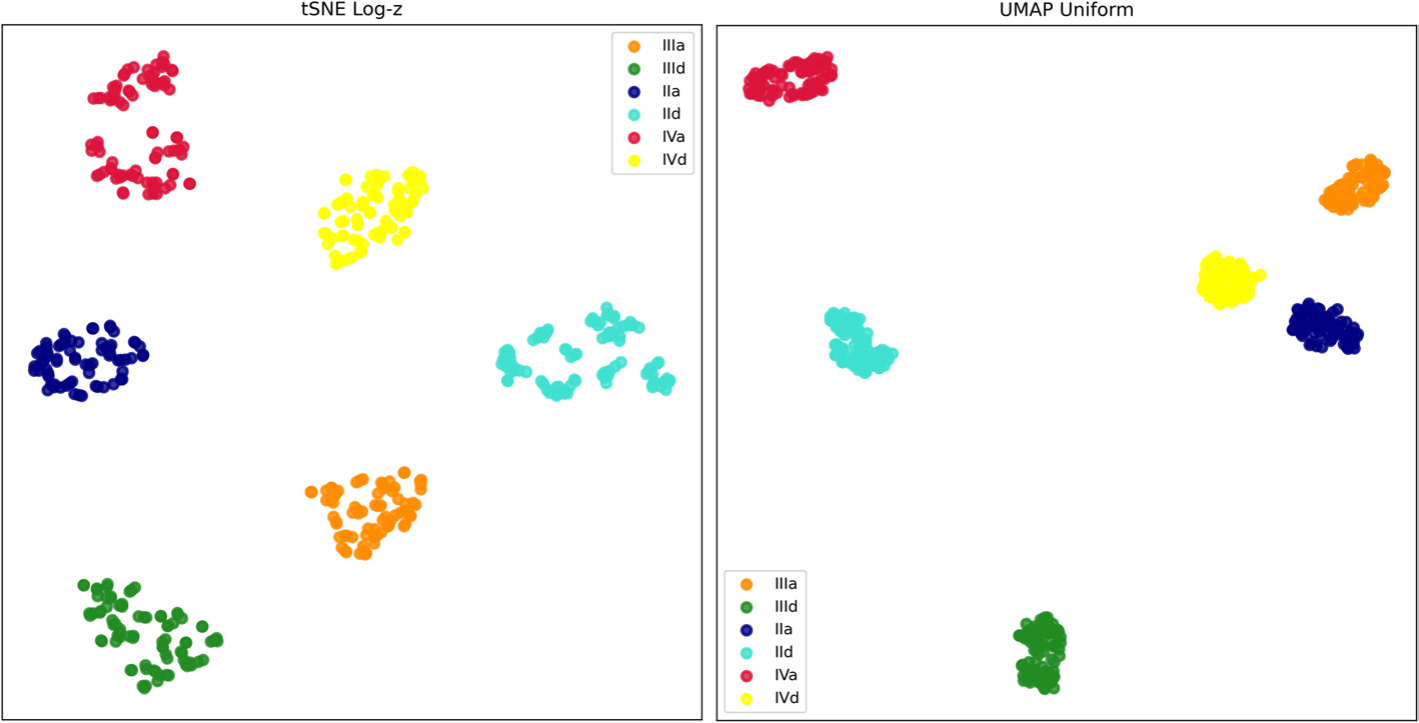
GED full embedding generating prognostic maps using tSNE Log-z values (on the left), and Uniform UMAP transformation (on the right)

**Fig. 7:**
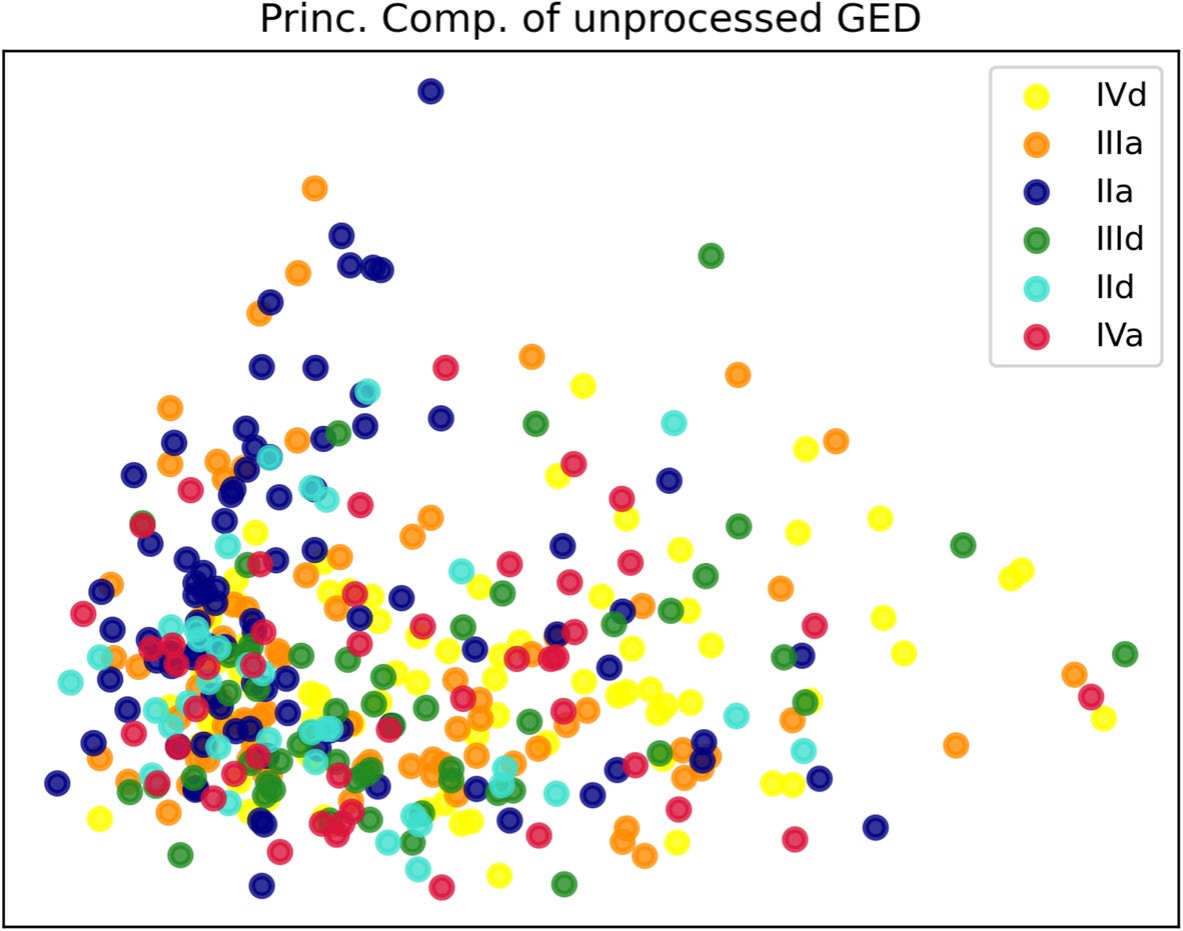
The scatterplot displays the first two principal components of $\log _{2}$ expression levels for the hub and seed genes before preprocessing. Total explained variance is $63.8\%$

In both experimental conditions, values of optimal t-SNE and UMAP configurations, together with clustering algorithms parameters and their internal scores, were aggregated into Tables 4 and 5. After parameter optimizations, clustering outcomes of the embeddings have been evaluated by external clustering metrics as a final assessment. Complete GED embedding reached a score of 1 in all external evaluation metrics for t-SNE, and UMAP obtained the same score with Uniform transformation on all external criteria; Log-z and Normal transformations had all values above 0.9935. For example, Fig. 6 demonstrates the bi-dimensional embedded space of Log-z paired with t-SNE on the left and UMAP with Uniform preprocessing on the right. Both panels create well–defined groups of patients and data transformation returns quickly interpretable prognostic charts to support medical decisions. By comparison, the $log_{2}$ unprocessed GED bi-dimensional plane of the two components with maximal explained variance from the principal component analysis was plotted in Fig. 7.

**Table 4:** t-SNE summary table

		Parameters						Clust. Param.^a^				
Emb.	Transf.	$\theta$	EE	LR	Metr.	Perp.	Clust.	parameter 1	parameter 2	Sil.	CHI	DBI
Full	Log-z	0.35	20	17	corr	10	hdbscan	min cl s=50	min s=1	0.761	2831.66	0.343
Full	Unif	0.35	12	100	corr	10	hdbscan	min cl s=50	min s=1	0.805	3515.45	0.269
Full	Norm	0.57	16	50	cheb	25	birch	bf=5	th=0.2	0.463	600.45	0.774
Part.	Log-z	0.57	20	200	corr	25	birch	bf=54	th=0.73	0.417	601.63	0.781
Part.	Unif	0.57	24	25	cheb	20	birch	bf=80	th=0.26	0.416	549.99	0.801
Part.	Norm	0.57	20	1000	cheb	25	SC	neighbors=10	-	0.355	409.97	0.828

^a^*MIN CL S* smallest size grouping, *TH* threshold, *BF* branching factor, *MIN S* minimal samples

**Table 5:** UMAP summary table

		Parameters										Clust. Param.^a^				
Emb.	Transf.	LR	LC	Metr.	MiD	NN	NSR	RS	Mix	Sp	Clust.	parameter 1	parameter 2	Sil.	CHI	DBI
Full	Log-z	0.1	1	mink	0.2	8	7	2	0.5	0.25	AP	pref=-34.3	damp=0.714	0.619	2686.95	0.505
Full	Unif	10	1	hamm	0.5	50	5	1	0.25	4	mb k-m	bat s=10	-	0.836	13263.93	0.246
Full	Norm	10	4	hamm	0.2	15	7	1	0.75	0.25	SC	$\gamma$=10	-	0.602	1302.76	0.577
Part.	Log-z	5	1	hamm	0.05	50	9	2	0.1	2	birch	bf=13	th=0.2	0.601	1151.23	0.495
Part.	Unif	0.1	1	hamm	0.01	30	5	3	0.1	0.25	AP	pref=-34.3	damp=0.71	0.729	3384.71	0.414
Part.	Norm	0.1	1	hamm	0.2	50	5	3	0.25	1	birch	bf=78	th=0.2	0.541	936.61	0.598

^a^*PREF* preferences for each point, *BAT S* size of the mini batches, *DAMP* damping factor, *TH* threshold, $\gamma$ kernel coefficient of radial basis function

**Fig. 8:**
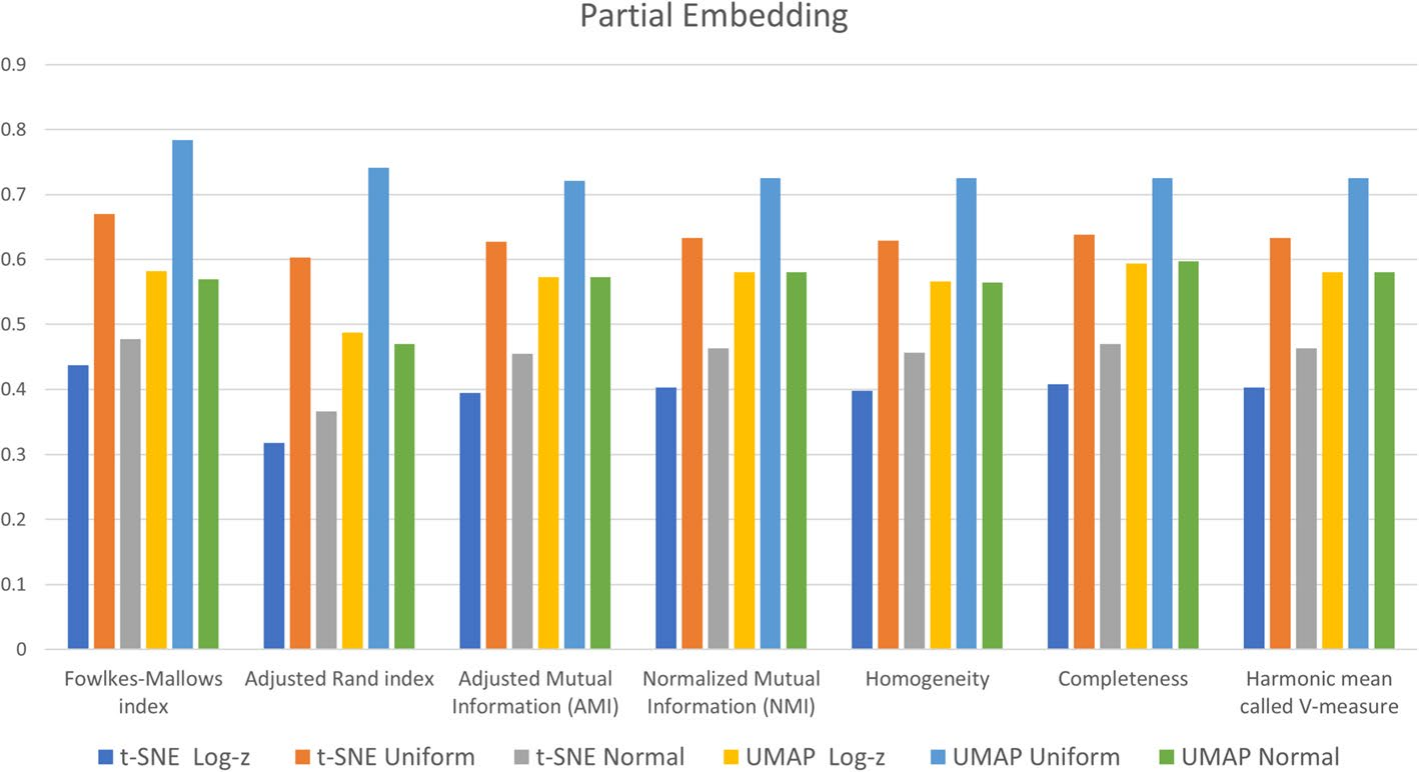
External evaluation metrics on partially embedded data

External scores during partial embedding were included in Fig. 8: Uniform distribution mapping inserted in a “double discretization” pipeline shows better outcomes than Log-z (single discretization stage) and Normal mapping both at t-SNE and UMAP. This observation is confirmed by performance measured with external indexes between full and partial embedding, appearing as a percentage of decay in Table 6.

**Table 6:** External evaluation metrics

	t-SNE			UMAP		
Metric	Log-z	Unif	Norm	Log-z	Unif	Norm
Fowlkes-Mallows index	-56.3	-33.0	-52.3	-41.5	-21.6	-42.9
Adjusted Rand index	-68.2	-39.7	-63.3	-51.0	-25.9	-52.8
Adjusted Mutual Information	-60.6	-37.3	-54.5	-42.3	-27.9	-42.3
Normalized Mutual Information	-59.7	-36.7	-53.7	-41.6	-27.5	-41.6
Homogeneity	-60.2	-37.1	-54.4	-43.0	-27.5	-43.2
Completeness	-59.2	-36.2	-53.0	-40.2	-27.5	-39.9
Harmonic mean (V-measure)	-59.7	-36.7	-53.7	-41.6	-27.5	-41.6

### Parameter space exploration

In this section, t-SNE and UMAP parameters applied to calculate dimensionality reduction during the complete embedding condition underwent a sensitivity analysis to determine the impact of algorithm parameters on clustering outcomes. The “metric” parameter has been transformed into scalar ordinal values for this investigation. Silhouette coefficient was selected as a concise measure of clustering appraisal (response variable) while scanning the configuration of parameters that optimizes data fitting. Traditionally parameter evaluation could be examined through linear regression to find non-deterministic linear relationships between parameter values [52]. In the current study, the nature of manifold-based dimensionality reduction poses the challenge of a nonlinear situation. For this reason, we reformulated the problem in terms of predictive performance by employing ensemble regression techniques to find the combination of t-SNE or UMAP parameters that maximizes clustering outcomes. During this phase, parameters of the regressors were left in their standard configuration to avoid regressor-specific optimization that could add a source of bias in the comparisons. The experimental setup could be exemplified by a table, with rows containing all the available combinations of parameters previously acquired while columns represent the possible combinations of parameters. Combinations of parameters could range from 2 to 5 for t-SNE (26 in total) and 2 to 9 for UMAP (502 in total). Parameter values previously collected, whose number is included under “available combinations” in Tables 7 and 8, were initially subdivided into the train (75$\%$) and test sets (25$\%$). The train set went through a 5-fold cross-validation for regressor selection, while the test set was employed to estimate the importance of the t-SNE or UMAP parameters. Eight nonlinear regressors were validated, and the best one was subsequently tested over each discretization pipeline’s parameter combinations. Tables 7 and 8 provide the parameter subset with the corresponding top $R^{2}$ score (also called the coefficient of determination) at the test set. In the context of this GED investigation, regression analysis suggests that fine-tuning parameters of UMAP seem crucial because most of them contribute to the clustering goodness of fit. This observation also confirms the findings of other authors on UMAP usage for GED data analysis [53]. Regarding t-SNE parameters, Metric and Perplexity are shared among different pipelines indicating their relative importance.

**Table 7:** t-SNE Parameter space exploration

	Available	Reduced parameter set			All 5 parameters	
Pipeline	Combinat.	R$^2$	Selected parameters	Regressor	R$^{2}$	Regressor
Uniform	615	0.968	$\theta$, LR, Metr, Perp	ETR^a^	0.961	ETR
Log-z	601	0.835	Metr, Perp	Bagging	0.814	ETR
Normal	608	0.884	LR, Metr, Perp	Voting^b^	0.821	ETR

^a^Meta estimator fitting 100 randomized decision trees

^b^Averaged individual predictions of Bagging, Random Forest and Gradient Boosting regressors

**Table 8:** UMAP Parameter space exploration

	Available	Reduced parameter set			All 9 parameters	
Pipeline	Combinat.	R$^2$	Selected parameters	Regressor	R$^2$	Regressor
Uniform	2173	0.825	LR,LC,Metr,MiD,NN	HGBR^a^	0.820	HGBR
			RS,Mix,Sp			
Log-z	2170	0.825	LR,LC,Metr,MiD,NN	HGBR	0.825	HGBR
			RS,Mix,Sp,NSR			
Normal	2173	0.810	LR,LC,Metr,MiD,NN	HGBR	0.803	HGBR
			RS,Mix,Sp			

^a^Histogram-based Gradient Boosting Regression Tree

### Classification using discretized data versus unprocessed GED

Prognosis prediction is still considered a challenge in bladder cancer [54]. In this machine learning experiment, a random forest classifier has been employed to determine the classification accuracy in discriminating patients based on the six labels that sum up tumor stage and survival (‘IIa’, ‘IId’, ‘IIIa’, ‘IIId’, ‘IVa’, ‘IVd’). The random forest classifier was already applied successfully in genomics [55–57], with profitable results also on imbalanced data [58]. The labels produced by each pipeline were employed to score results for the preprocessed data, while the ground truth labels were used for the raw GED. The hyperparameters of the classifier have been optimized by Bayesian optimization [59]. This technique explores the hyperparameter space of the classifier by adopting a gaussian process [60] that evaluates an objective function fitted for all combinations of hyperparameters, intending to exclude combinations that do not improve the classifier’s performance. The classifier’s parameters that underwent tuning were the number of trees in the forest, the maximal depth of each tree, the minimal number of instances needed to split a node or to determine leaf nodes, and the number of features to determine the best splitting. All experiments were carried out with a nested 5–fold stratified cross-validation with accuracies of the outer loop shown in Table 9, while the inner loop served for hyperparameter tuning. The random forest was programmed to account for class imbalance, weighting the input samples with stratification based on $\frac{tot.\,samples}{tot.\,classes\,\times \,class\,samples}$. This numerical experiment reported that discretization pipelines produce values that ensure higher accuracies compared to the $log_{2}$ raw GED from the original dataset. A Dummy classifier has been included as a baseline measure of chance level accuracy. The random forest classifier using $log_{2}$ unprocessed GED reaches an average balanced accuracy of $26\%$, while data preprocessed by discretization achieved more than double this value. As a final remark, the balanced accuracies of the labels obtained by each preprocessing pipeline on full GED embedding compared to the ground truth accomplished $100\%$ in all except the UMAP with Uniform transformation ($99.79\%$).

**Table 9:** Random Forest and Dummy classifiers balanced accuracy of preprocessed GED with discretizations pipelines vs. $log_{2}$ GED (accuracies are expressed as percentages)

Pipeline	RF Bal. Acc.	Dummy Bal. Acc.
tSNE Uniform	$61.4\pm 9.4$	$17.6\pm 3.4$
tSNE Log-z	$58.4\pm 9.3$	$17.4\pm 3.9$
tSNE Normal	$62.5\pm 9.0$	$13.9\pm 2.8$
UMAP Uniform	$61.4\pm 9.4$	$16.9\pm 2.8$
UMAP Log-z	$58.5\pm 8.5$	$13.3\pm 3.5$
UMAP Normal	$62.5\pm 9.0$	$14.2\pm 2.3$
$log_{2}$ GED	$26.0\pm 3.1$	$17.7\pm 4.5$

### Genes functional relevance

**Fig. 9:**
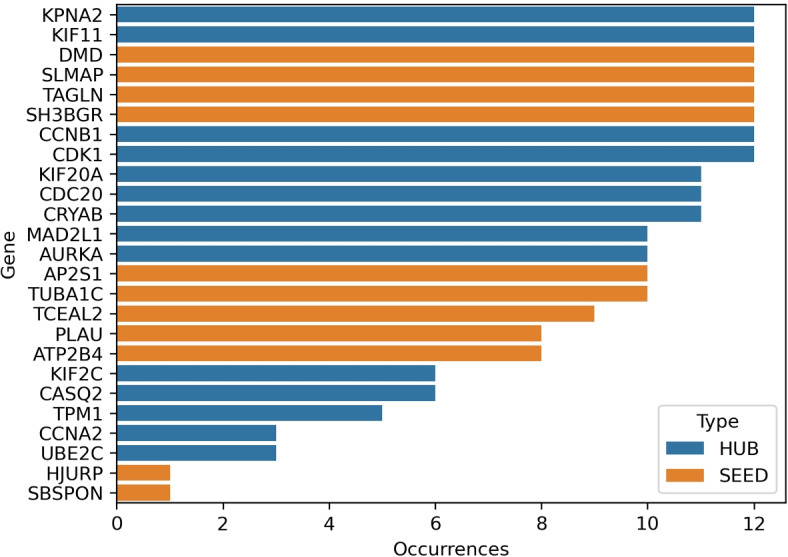
Barplot of gene relevance in categorizing the prognosis of the patients (agreement between RFECV and PI methods)

Two methodologies were employed to score the genes’ influence in predicting tumor stage and survival. It could be possible that genes of the set pre-selected by the database authors might have different involvement in the pathological status of the patients; thus, they could be evaluated concerning their importance in determining the disease outcome. Using the labels obtained during the complete embedding by the single and double discretization pipelines with t-SNE or UMAP and a forest of trees classifier, the genes after preprocessing (Fig. 5) were ranked by permutation importance [61, 62] (i.e., PI) and ulteriorly confirmed by recursive feature elimination [63, 64] with cross-validation (i.e., RFECV). These operations evaluated if genes could be rated relevant or not in determining class membership from the classifier’s scores. A random forest classifier was chosen as an estimator due to its popularity in statistical genetics, as reported in [62]; recently, it has also been used as a baseline classifier in [65]. The random forest classifier assumed class weights to compensate for their imbalance. Table 10 collects the 5–fold stratified cross–validation balanced accuracy obtained by the Random Forest classifier employed to score gene importance: accuracies reported are those on the subset of genes identified by PI or RFECV. The Dummy classifier, a baseline classifier acting as a reference for the chance level, has been included in Table 10. Random Forest accuracies were fairly above the chance indicated by the Dummy classifier, ensuring the safe application of the procedure. Table 11 collects the number of occurrences for each gene selected by PI and RFECV using the six pipelines of the complete embedding experiment. Genes with values equal to six were present as most influential over all pipelines. The last column of Table 11 sums up the total number of times a gene was ranked important by PI and RFECV: including two scoring methods, PI and RFECV, ensures a consensus in selecting relevant genes. Four hub (KPNA2, KIF11, CCNB1, CDK1) and four seed genes (DMD, SLMAP, TAGLN, SH3BGR) gather the largest consensus, being selected by both PI and RFECV methods throughout all analysis pipelines. Included in almost all occurrences are also the hub genes KIF20A, CDC20, and CRYAB. The relevance of each gene derived from the last column of Table 11 has been plotted as a barplot in Fig. 9. In the barplot, genes were ranked in ascending order.

**Table 10:** Random Forest balanced accuracies during gene relevance investigation (as percentages)

Pipeline	RF PI	RF RFECV	Dummy PI	Dummy RFECV
tSNE Uniform	$57.7\pm 10.9$	$61.3\pm 10.5$	$18.3\pm 3.5$	$16.3\pm 2.1$
tSNE Log-z	$54.2\pm 7.0$	$58.4\pm 8.2$	$16.6\pm 1.4$	$16.5\pm 0.5$
tSNE Normal	$53.2\pm 8.8$	$62.5\pm 8.8$	$15.3\pm 2.1$	$17.5\pm 1.9$
UMAP Uniform	$57.7\pm 10.5$	$61.4\pm 10.5$	$15.5\pm 4.9$	$19.3\pm 2.2$
UMAP Log-z	$56.5\pm 8.9$	$58.5\pm 8.9$	$16.5\pm 3.3$	$16.9\pm 3.9$
UMAP Normal	$53.2\pm 8.8$	$62.5\pm 8.8$	$18.9\pm 1.5$	$16.4\pm 5.4$
Average	$55.4\pm 9.1$	$60.7\pm 9.3$	$16.8\pm 3.3$	$17.2\pm 3.3$

**Table 11:** Occurrencies of the gene ranked most important by the six pipelines. Last column sums the number of times genes were top ranked by both PA and RFECV procedures

Gene	Type	Occur. PI top ranked	Occur. RFECV top ranked	Tot. occur. top ranked
KPNA2	HUB	6	6	12
KIF11	HUB	6	6	12
DMD	SEED	6	6	12
SLMAP	SEED	6	6	12
TAGLN	SEED	6	6	12
SH3BGR	SEED	6	6	12
CCNB1	HUB	6	6	12
CDK1	HUB	6	6	12
KIF20A	HUB	5	6	11
CDC20	HUB	6	5	11
CRYAB	HUB	6	5	11
MAD2L1	HUB	4	6	10
AURKA	HUB	4	6	10
AP2S1	SEED	4	6	10
TUBA1C	SEED	4	6	10
TCEAL2	SEED	3	6	9
PLAU	SEED	2	6	8
ATP2B4	SEED	2	6	8
KIF2C	HUB	1	5	6
CASQ2	HUB	0	6	6
TPM1	HUB	0	5	5
CCNA2	HUB	0	3	3
UBE2C	HUB	0	3	3
HJURP	SEED	0	1	1
SBSPON	SEED	0	1	1

## Discussion

Generally, discretization transforms features closer to a knowledge-level representation than continuous data [66]. In the current investigation, three discretization pipelines were merged with tree embedding and manifold reduction to check which experimental sequence could discriminate six groups of patients related to tumor stage and survival in bladder cancer. Full data embedding with decision trees paired with t-SNE or UMAP dimensionality reductions build bi-dimensional data representations with dense and well-separated point clouds. For instance, both t-SNE and UMAP techniques are available in a recently released software app for GED visualization [67], confirming they are well-established visualization approaches in the omics disciplines. On the contrary, during a partial embedding experiment simulating the addition of new patient data to an existing tree model, only Uniform transformation with UMAP maintains a certain proportion between GED from new patients ($25\%$) and performance (metric scores ranged between $21.6\%$ and $27.5\%$). Furthermore, results on non-linear reduction techniques using partially embedded data showed that t-SNE behavior is less efficient than UMAP as measured by external clustering validation metrics. These findings get support from recent trends in literature that exploit UMAP methodology to display genetic interactions [68], and gene variability [69]. The UMAP superiority is also confirmed by the outcomes of the internal validation coefficients (Tables 4 and 5) with higher silhouette and CHI coefficients and lower DBI. However, according to the parameter space examination, multiple UMAP parameters play an essential role in the outcomes of dimensionality reduction, thus requiring a procedure that searches for the best combination. The drawback is that parameter space search could be time-consuming, especially on off-the-shelf hardware.

Medical doctors may not be acquainted with machine learning techniques; consequently, an effective tool for GED interpretation might enhance the visual understanding of multi-dimensional GED datasets (we also called them prognostic maps). Research presented by this study focuses on 2D t-SNE and UMAP reduction because we explored the possibility of producing discernible patterns in the data that summarize the disease progression in patients. Similar to other works in the literature, this investigation adopts an intermediate step to transform GED data distributions by decision tree embedding. This phase should pull out interesting characteristics in the input samples that are otherwise not directly observable. For example, pathways of associated genes or gene pairs with combined effects may be reflected by high correlations or network attributes [70]. A different type of embedding could be obtained with autoencoders to learn structures in the data by exploiting deep architectures. In the intermediate layers of autoencoders, dimensionality is diminished to achieve a more dense representation of the data. This possibility has been examined by [71] or [72]. Other authors also suggest the application of graph embeddings (also called network representation learning) for GED transformation [73] to map nodes and edges of the gene network while preserving their properties and information.

The benefit of discretization was demonstrated by a supervised machine learning experiment that tried to classify the six classes of outcomes from the raw or preprocessed GED. While the original dataset resulted in a balanced accuracy slightly above the chance level ($26\%$ versus a chance level of $17.7\%$ exemplified by the Dummy classifier), discretized data obtained higher results. Moreover, a little difference was found between the accuracies of the double discretization pipelines ($61.4\%$ and $62.5\%$) compared to the single discretization pipeline involving Log-z ($58.5\%$), with marginally higher accuracies in the former case. The difficulties in classifying the original GED dataset might also be related to the high correlation between specific genes, as seen in Fig. 3. In machine learning, correlated features might be suboptimal to solve classification tasks as they provide little extra information. The transformations of the GED during the preprocessing phase probably improved this aspect, also verified during the numerical experiments in [7] on the same dataset for binary classification.

The relevance characterization using relative gene importance identified a subset of genes by their prominence in defining tumor stage and patient survival. Results of this machine learning experiment are included in Table 11. Eight genes collected the highest consensus and were ranked most relevant by the proposed analysis pipelines. Literature confirms the importance of the top-ranked genes as KPNA2, recently identified as involved in cancer progression in several studies [74–76], or KIF11 [77, 78]. The seed gene DMD seems more related to survival [79], while the transgelin gene TAGLN is closely connected to oncogenic transformation and, consequently, prognosis in bladder cancer patients [80, 81]. Another top-ranked gene was SH3BGR, a family of genes that might indicate a low survival rate in bladder cancer in its subtype SH3BGRL3 [82]. Gene CCNB1 seems related to aggressive forms of bladder cancer and cell proliferation [83], while the cyclin-dependent kinase CDK gene could be related to bladder tumor staging and prognosis [84–86]. Apart from the top-ranked eight genes, three other genes were included as most influential by nearly all pipelines. They were KIF20A, a gene inducing proliferation [87], CDC20, which might be connected with radio-resistance, thus survival [88, 89], and CRYAB [90–92], whose overexpression was mentioned in cancer signaling pathways.

### Estimated computational times

Table 12 contains the computational times of each step of the experimental pipelines for the complete data embedding. The average computational times of the dimensionality reduction phase should be multiplied by the t-SNE or UMAP total number of parameters investigated to get the total time spent in this step of the elaboration. Similarly, the table reports the tuning time of the clustering algorithms as an average of the six algorithms tested. Indeed, clustering is considered an “explorative” analysis requiring the evaluation of the results from different methods and tuned parameters. For example, the estimated computational times for investigating the parameter space of UMAP with the Normal preprocessing pipeline were approximately 282.15 hours, while the t-SNE pipeline took 36.86 hours. In addition, the total time should be doubled to include the computations needed during the partial embedding experiment. All numerical experiments were carried out on commodity hardware (laptop computer with an i5 10th generation processor and 16Gb RAM).

**Table 12:** Average computational times (in seconds) for each single operation performed in the analysis pipeline during the complete experimental embedding

Pipeline	Forest Emb.	Dim. Red.	Clustering	Param. Comb.
tSNE Uniform	$7.17\pm 1.38$	$6.95\pm 0.55$	$34.14\pm 35.18$	615
tSNE Log-z	$7.16\pm 1.61$	$10.18\pm 8.65$	$33.39\pm 36.82$	601
tSNE Normal	$6.46\pm 1.38$	$9.54\pm 8.89$	$33.71\pm 37.63$	608
UMAP Uniform	$3.47\pm 1.12$	$8.15\pm 11.92$	$151.25\pm 305.05$	2173
UMAP Log-z	$3.72\pm 1.74$	$13.04\pm 15.78$	$73.27\pm 128.05$	2170
UMAP Normal	$4.19\pm 1.39$	$3.59\pm 0.67$	$76.61\pm 131.14$	2173

## Conclusion

This study evaluated if GED discretization approaches could be integrated into a new analysis pipeline extending patient identification by tumor stage and survival. Complete data embedding created precise prognostic maps suitable for data-driven medical decision-making. In a second numerical experiment using partially embedded data to simulate new patients’ inclusion in the model, performance seems stable only applying the Uniform double stage discretization sequence and UMAP non-linear reduction. Findings on both experimental conditions support using the UMAP technique in omics data analysis as emerging in recent literature on the same topic. However, a further investigation of UMAP parameter space did not identify a significant subset of relevant parameters to consider for speeding up algorithm tuning. This situation underlines the importance of adjusting multiple UMAP parameters for precision medicine studies. A machine learning procedure to establish gene importance in determining six classes of outcomes has been demonstrated through feature permutation or recursive feature elimination. Through this methodologies, a subset of relevant genes for bladder cancer prognosis has been identified. Another machine learning experiment showed how the classification of patients using the preprocessed data with single or double discretization pipelines achieved higher accuracy than unprocessed data. The numerical experiments in the current investigation testing three distinct preprocessing sequences based on single or double discretizations helped to discriminate more effectively six possible patients’ outcomes given a bladder cancer GED dataset from a cross-sectional study.
